# Ethical deficits in poor physician leadership: a deductive content analysis of virtue ethics, consequentialism and deontological ethics

**DOI:** 10.1108/LHS-02-2026-0036

**Published:** 2026-09-08

**Authors:** Sari Huikko-Tarvainen, Kimmo Tarvainen

**Affiliations:** Faculty of Medicine, Oulu University, Oulu, Finland; Department for Work and Gender Equality, Ministry of Social Affairs and Health, Tampere, Finland

**Keywords:** Physician leadership, Virtue ethics, Consequentialism, Deontological ethics, Healthcare

## Abstract

**Purpose:**

This study aims to examine how virtue ethics, consequentialism and deontological ethics are violated in poor physician leadership.

**Design/methodology/approach:**

Semi-structured interviews with 50 physicians across all career stages were analysed using deductive content analysis based on a normative ethical framework of virtue ethics, consequentialism and deontological ethics.

**Findings:**

The results revealed leadership behaviours largely contradictory with all three normative ethical frameworks. Regarding virtue ethics, informants described deficits in honesty, fairness, compassion, integrity and responsibility. From a consequentialist perspective, leadership practices led harmful to outcomes, including psychological strain, reduced trust and well-being, impaired organizational functioning and negative implications for patient care. In terms of deontological ethics, informants noted violations of duties, moral rules and respect for persons, including inequitable treatment, discriminatory decisions, breaches of procedural justice and failures in supervisory responsibilities.

**Practical implications:**

Across the normative ethical frameworks, the findings revealed an absence of conduct consistent with normative moral principles. These results highlight the ethical fragility of leadership practices within the studied health-care context and underscore the need for strengthened ethical grounding in leadership development and organizational governance. These findings can be used to learn and to improve ethical decision-making in physician leadership. Cultural or health-care contexts differences may limit the transferability of the findings.

**Originality/value:**

No prior empirical research has specifically examined how normative ethics is violated in poor physician leadership.

## Introduction

Leadership within health-care environments is a key factor influencing both the well-being of health-care professionals and organizational outcomes (Clay-Williams *et al.*, 2017). Physician leadership plays an essential role in promoting a culture of high-quality patient care, strengthening team collaboration and supporting the personal and professional growth of the medical workforce (Clay-Williams *et al.*, 2017; Kaiser *et al.*, 2020). Leadership styles in health-care significantly shape employees’ perceived agency (Günzel-Jensen *et al.*, 2018), and overall quality of life is closely tied to the quality of leadership (Antonakis, 2011). Much of the literature on effective leadership highlights its inherent moral dimension, noting that leaders typically strive for positive outcomes (Ciulla, 2005; Clements and Washbush, 1999).

Nevertheless, leaders may at times behave unethically (Bass, 1998). Addressing these concerns is essential, as poor leadership can appear in various forms that critically undermine organizational functioning and generate serious negative consequences, including increased turnover (Aasland *et al.*, 2010; Huikko-Tarvainen *et al.*, 2022). Despite this, the issue of misconduct in physician leadership has received limited attention (Huikko-Tarvainen *et al.*, 2022). Behaviours such as micromanagement, emotional unavailability and public humiliation have been shown to cultivate fear and mistrust, ultimately damaging staff well-being and patient care (Huikko-Tarvainen *et al.*, 2022). In addition, the conduct of physician leaders affects the well-being and burnout levels of the physicians they supervise (Shanafelt and Noseworthy, 2017).

The dark-side perspective addresses the positivity bias in leadership research and offers guidance for enhancing team, organizational and national performance by reducing the likelihood of appointing unsuitable individuals to leadership positions and by fostering greater self-awareness and self-regulation among leaders (Hogan *et al.*, 2021). Professional integrity and intellectual rigor demand that the examination of what is harmful accompanies efforts to cultivate what is beneficial (Kellerman, 2024). Moral distress – experienced by both clinicians and leaders – can be mitigated through transparency and value alignment (Hertelendy *et al.*, 2022), while its roots in organizational and systemic conditions further underscore the need for active engagement of the physician voice to address these underlying contributors (Locke *et al.*, 2025).

While ethical leadership is generally regarded as beneficial, some scholarship suggests that its interpretation is shaped by differing moral philosophies and cultural and organizational contexts, although other scholars argue that underlying cross-cultural ethical principles provide a shared foundation for ethical leadership (Eisenbeiss, 2012). Research on organizational and institutional failure further indicates that adverse leadership outcomes cannot be explained solely by individual characteristics or leader behaviour. Rather, such failures emerge from the interaction of organizational culture, management systems, structural conditions, regulatory environments and broader institutional pressures that shape behaviour and decision-making over time (Hald *et al.*, 2021).

The present study aims to advance understanding of how virtue ethics, consequentialism and deontological ethics are violated in poor physician leadership by examining the lived experiences of physicians across different hierarchical levels.

### The centrality of ethics in the medical profession

Medicine integrates scientific training with a strong moral foundation, with societal trust as its cornerstone. Physicians maintain expertise, autonomy and public confidence by upholding ethical duties such as competence, respect for human values and commitment to social justice. Medicine is thus a moral practice oriented towards patients’ welfare, dignity and trust (Ribeiro Ferreira *et al.*, 2025).

Medical ethics guides morally challenging decisions and daily clinical practice. Historically rooted in the Hippocratic tradition, it emphasizes patient welfare, confidentiality, and competent care (Finnish Medical Association, 2021). Modern developments – technological advances, organizational pressures and evolving institutions – reinforce ethics as a stabilizing guide, while patient autonomy, empowerment and cultural pluralism complement traditional standards (Salloch *et al.*, 2016). Ethics shapes career-long decisions, sustaining societal trust and preventing professional exhaustion (Finnish Medical Association, 2021).

While law defines practice boundaries, ethics articulates normative values; adherence is essential when conflicts arise (Finnish Medical Association, 2021). Core principles – beneficence, non-maleficence, respect for dignity and autonomy and justice – support professional virtues such as honesty, compassion, integrity and altruism, enabling physicians to serve patients and society (Finnish Medical Association, 2021; Unger *et al.*, 2020). Furthermore, ethical obligations form the social contract. Physicians are granted licensure and societal trust based on their specialized knowledge and skills, in exchange for a commitment to prioritize patients’ interests, act with integrity and provide care with competence and altruism. (Finnish Medical Association, 2021). Accordingly, physicians are expected to be well-versed in ethics, which constitutes a foundational element of the medical profession.

### Ethical aspects of physician leadership

Ethical aspects of physician leadership are present at every hierarchical level of the medical profession, and ethical leadership is regarded as a central element of effective physician leadership (Huikko-Tarvainen, 2022). Physician leaders are expected to exemplify ethical standards through their work and to act in accordance with the principles they endorse (Brown and Treviño, 2006). Ethical leadership is expressed both through external direction and an internal sense of responsibility (Bowman and Swanwick, 2017). Ethical leaders present themselves as honest, compassionate and principled in their approach to impartial decision-making. They establish ethical norms and conduct their work in accordance with these expectations (Brown and Treviño, 2006). They do not show favouritism or rely on distrust to gain power or pursue personal aims, as they must promote ethical conduct and prevent unethical behaviour.

Ethical leaders are viewed as capable of creating reciprocal trust and respect among subordinates, resolving disputes between different parties and guiding subordinates to recognize the importance of adaptive problem-solving that supports long-term well-being. Yukl, (2013). They treat their followers with equal dignity and recognition and uphold principles of what is right and good (Yukl, 2013). Leaders articulate a vision that incorporates subordinates’ ideas, values and needs, and they accept personal risks and take action to realize that vision (Yukl, 2013).

## Theoretical framework

### Normative ethics

Normative ethics – including virtue ethics, consequentialism and deontological ethics (Doukas *et al.*, 2022; Everett *et al.*, 2018; Tseng and Wang, 2021) – provides distinct yet complementary perspectives for analysing professional conduct, leadership and organizational practices in health care.

### Virtue ethics

Virtue ethics grounds moral appraisal in the character traits and dispositions of moral agents rather than in rules or outcomes (Doukas *et al.*, 2022; Malik *et al.*, 2020). Contemporary medical virtue ethics highlights professional virtues (e.g. honesty, compassion, integrity) and the pedagogical role of moral exemplars (Doukas *et al.*, 2022), while emphasizing the centrality of phronesis (practical wisdom) as the integrative virtue that guides context-sensitive judgement (Conroy *et al.*, 2021; Malik *et al.*, 2020). Empirical studies and scoping reviews suggest that virtue-ethical concepts have been applied in medical education and professional formation to support humanistic practice and ethical deliberation beyond reliance on prescriptive checklists (Conroy *et al.*, 2021; Doukas *et al.*, 2022; Malik *et al.*, 2020). Virtue-ethical approaches thus highlight the cultivation of professional character and phronesis as essential elements of sound clinical judgement and humane care (Conroy *et al.*, 2021; Malik *et al.*, 2020).

### Consequentialism

Consequentialism holds that the moral status of actions depends on their outcomes, typically assessed in terms of welfare, preference satisfaction or other outcome metrics (Häyry, 2021). Social psychological research indicates that people often infer moral character differently depending on whether decisions involve instrumental harm or impartial beneficence (Everett *et al.*, 2018). The literature on consequentialist reasoning accordingly emphasizes both the trade-offs among competing outcomes in applied contexts (Card and Smith, 2020), and the social consequences of instrumental harm, including its effects on trust and reputational judgements (Everett *et al.*, 2018).

### Deontological ethics

Deontological theories evaluate moral rightness based on adherence to duties and rules rather than consequences (Donaldson, 2017; Tseng and Wang, 2021). In medical ethics, Kantian deontology emphasizes respect for persons and duty-guided action, with contemporary discussions further highlighting the role of Kantian autonomy in clinical contexts (Elsner and Rampton, 2022). Deontological reasoning thus prioritizes duties and rights rather than consequences and provides an ethical reflection on autonomy and moral responsibilities in clinical contexts (Donaldson, 2017; Elsner and Rampton, 2022; Tseng and Wang, 2021). By underscoring the intrinsic respect owed to persons, Kantian formulations continue to inform discussions on ethical decision-making in medicine, particularly when contrasted with outcome-driven approaches (Donaldson, 2017; Elsner and Rampton, 2022; Tseng and Wang, 2021).

## Method

This research used a qualitative design, conducting semi-structured interviews with 50 physicians across different hierarchical positions (residents/physicians in specialist training, specialists, department heads and chief physicians) within the Central Finland Health Care District during April – June 2017 and July – August 2018. The study was conducted among 485 doctors in the area. Participation was voluntary, with invitations distributed via internal email. Participants received information about the study, including their right to withdraw at any time. No incentives were offered. Interviews were conducted individually, typically in participants’ offices, and lasted 11–85 min (approximately 25 h in total). All interviews were audio-recorded, transcribed verbatim, anonymized and prepared for coding, resulting in 619 A4 pages (Calibri, 12-point, single-spaced). The interview guide included questions designed to examine physician leadership from multiple perspectives and to allow informants to share their personal experiences of physician leadership. An English translation of the original Finnish interview guide is provided in the Appendix. For the present study, only data relating to poor leadership were analysed; data on other themes have been published previously. The primary aim was to examine: “How normative ethics (virtue ethics, consequentialism and deontological ethics) are violated in cases of poor physician leadership?”

### Deductive content analysis

Given the presence of a well-established, theory-driven normative ethical framework, deductive content analysis was used. Interview data were analysed using a pre-existing framework derived from normative ethical theory (virtue ethics, consequentialism and deontological ethics). Deductive content analysis is an established method for systematically evaluating qualitative data against predefined theoretical categories and is appropriate when existing theory or normative constructs guide the investigation, allowing researchers to assess the presence, absence or variation of theoretically relevant phenomena across empirical data (Eriksson and Kovalainen, 2008; Kyngäs and Kaakinen, 2020). The analysis followed established guidelines for directed deductive content analysis (Kyngäs and Kaakinen, 2020). Based on theoretical definitions of virtue ethics, consequentialism and deontological ethics (as detailed in the theoretical framework), a codebook was created specifying categories: virtue-ethical violation, consequentialist violation and deontological violation. More specifically, in deductive coding, virtue-ethical alignment (Doukas *et al.*, 2022; Malik *et al.*, 2020) was defined by behaviours and practices that exemplify recognized professional virtues, such as transparent communication, empathetic supervision and equitable treatment. Violations of virtue ethics were defined as behaviours that manifest vices or reflect an absence of virtues – such as deception, public humiliation or neglect of subordinate welfare – thereby undermining character-based professional formation.

Next, consequentialist alignment (Card and Smith, 2020; Everett *et al.*, 2018) was operationalized as decision-making (explicit or implicit) aimed at producing better aggregate outcomes for relevant stakeholders, including patients, staff and the organization. Conversely, behaviour that systematically causes harm to stakeholders without apparent consideration of overall welfare was coded as acting against consequentialist norms. The literature on consequentialist trade-offs and social inferences from instrumental harm guided attention to secondary effects, such as erosion of trust and reputational costs.

Finally, deontological alignment (Donaldson, 2017; Tseng and Wang, 2021) was identified when actions demonstrably respect duties and persons, for instance through due process in decision-making, equitable contractual practices and apology or reparative measures. Violations of deontological ethics were indicated by instrumentalization, procedural breaches, unequal treatment or failure to fulfil supervisory duties owed to colleagues and patients. All interview transcripts were read repeatedly to develop a thorough understanding of the content, context, and variation in participants’ accounts. Each transcript was systematically coded using the predefined categories. Relevant segments of text (meaning units) were identified and assigned to one or more categories when they aligned with the principles of normative ethics. Following initial coding, codes were grouped into broader analytic themes reflecting patterns across informants. These themes corresponded to practices and behaviours judged as ethically aligned or misaligned within the three frameworks. The final analytic output consists of a thematic structure summarizing how physicians’ accounts reflect deviations from the normative standards of virtue ethics, consequentialism and deontological ethics, illustrated with quotations from the data.

To enhance trustworthiness, the analytic process was documented in detail. Repeated readings of the data (familiarization), systematic coding, and transparent documentation of analytic decisions supported credibility and dependability (Graneheim and Lundman, 2004). Additionally, the analysis was conducted twice and subsequently reviewed by all authors to further strengthen trustworthiness. Throughout the coding and analysis phases, regular meetings were held among the authors to provide feedback and refine the coding framework. Reflexive discussions were maintained and inter-coder agreement was assessed (Kyngäs *et al.*, 2020). Researcher triangulation, in which multiple researchers examined the data and cross-verified interpretations and conclusions, further enhanced the validity and reliability of the findings (Eriksson and Kovalainen, 2008). Moreover, adherence to methodological rigor further reinforced the trustworthiness and reliability of the results (Braun and Clarke, 2019).

### Ethical approval

Ethical approval for the study was obtained from the Central Finland Care District officials. The research did not receive financial support from any public, commercial or non-profit organizations. Informed consent was obtained from all participants, ensuring they were aware of their rights, including the right to withdraw at any time without any consequence. Confidentiality was maintained by anonymizing all data and securely storing interview recordings and transcripts. This study was conducted in accordance with national and international ethical standards for non-medical research involving human participants, adhering to guidelines established by the Finnish National Board on Research Integrity and the data protection regulations of the European Union (Finnish National Board on Research Integrity TENK guidelines 2019, 2019).

### Strengths, limitations and future research

This study examines violations of virtue ethics, consequentialism and deontological ethics in poor physician leadership through a deductive content analysis of semi-structured interviews with Finnish physicians (*n* = 50) (Doukas *et al.*, 2022; Everett *et al.*, 2018; Tseng and Wang, 2021). The sample included physicians across hierarchical levels, ensuring diverse perspectives and strengthening the breadth and depth of findings. Systematic coding procedures and debriefing enhanced the rigor, credibility, and trustworthiness of the analysis Elo and Kyngäs, (2008). Despite these strengths, the study has limitations. The deductive design relies on interview data from a defined sample of self-reported experiences among Finnish physicians, which may limit transferability to other cultural or health-care contexts. Although the findings align with international leadership literature, generalizability may be constrained by specific organizational and cultural conditions. Future mixed-methods research could assess interventions combining leadership skills training, organizational justice measures and virtue-formation curricula, and their effects on staff well-being and patient-care outcomes. As the study predates COVID-19, which expanded leadership and management responsibilities beyond formal physician leadership roles (Rotenstein *et al.*, 2021), future research should examine whether post-pandemic contexts have altered these findings, although the qualitative evidence on ethical violations is not fundamentally time-sensitive.

## Results

The deductive content analysis revealed that poor physician leadership is characterized by behaviours that violate all three components of normative ethics: virtue ethics, consequentialism and deontological ethics. From a virtue ethics perspective, leadership was often marked by a lack of honesty, fairness, compassion and integrity. Informants described non-transparent decision-making, public humiliation, bullying, avoidance of responsibility and discriminatory practices, reflecting deficiencies in moral character and virtuous conduct. From a consequentialist perspective, these behaviours produced predominantly harmful outcomes. Psychological distress, diminished trust, impaired well-being and increased turnover were commonly reported. Informants also highlighted negative effects on organizational functioning and patient care arising from a lack of supervision and inadequate support for junior physicians. From a deontological perspective, the behaviours reflected clear violations of duties, moral rules and respect for persons. Physicians reported experiences of unfair treatment, inequitable salary practices, discriminatory hiring, gender-based disadvantage, breaches of procedural justice and failures to fulfil supervisory responsibilities.

Overall, the data indicate that poor physician leadership consistently contravenes the foundational principles of normative ethics. Specifically:

- Virtue ethics: Leadership lacked honesty, fairness, empathy, integrity and courage.
- Consequentialism: Actions produced negative outcomes, including psychological harm, turnover, mistrust and reduced workplace safety.
- Deontological ethics: Breaches of duties, moral rules, equality and respect for persons were evident.

In listing below, the findings are presented in greater detail and are accompanied by illustrative citations from informants’ responses.

Summary of the deductive content analysis of ethical deficits in poor physician leadership, categorized by virtue ethics, consequentialism and deontological ethics, with illustrative citations.

VIRTUE ETHICS (focus on character, virtues such as honesty, fairness, compassion, integrity):

- Lack of honesty and transparency (e.g. decisions made without communication), absence of compassion and empathy, failure of justice and fairness; avoidance of responsibility, bullying and humiliation; discriminatory behaviour.

*Lack of honesty and transparency*

*Manipulation of job descriptions without clear communication*:

- She was first told that she would be in the ward. Then it was said that she would also handle this, this, and this. (I13).

*Absence of compassion and empathy*

*Supervisors failing to provide emotional support after distressing events such as patient deaths or suicides*:

- When I personally experienced a patient’s death, I just left work gritting my teeth. (I24)

*Dismissive responses to physicians’ struggles*:

- I was struggling to cope, his [the leader] response was, ‘Me neither.’ That was the support I got. (I50)

*Leaders who didn’t want to listen and offered no support in difficult career situations*:

- I wanted to talk to my supervisor about a difficult situation in my career, … but he didn’t want to listen. (I39).

*Failure of justice and fairness*

*Inequitable workload allocation and exploitation of overburdened physicians*:

- They felt overworked and stuck in a salary pit compared to others with similar roles. The boss offered a one-euro raise and suggested they take on the maternity clinic as well. (I14)

*Unfair salary discussions, accompanied by aggression and lack of apology*:

- I went to discuss whether it would be possible for us to at least have the same base salary since we were doing the same job. … He [the leader] then tried to explain other salary issues but clearly wasn’t aware of the difference between base and total salary. I corrected him, and he got upset. (I15)

*Denial of earned vacation despite a long contract*:

- I had been working at one workplace for about two years, and my two-year contract was ending on August 31st … I was told I could take vacation in September. (I49)

*Favouritism in hiring*:

- The job posting is up on some notice board for two hours, and someone who’s already earmarked for the position is notified. (I29)

*Preferential recruitment benefiting spouses of desired specialists*:

- There’s this peculiar thing … If they want that specialist doctor, and the spouse is a doctor too, so they’ll find a position for the spouse as well. (I29)

*Gender discrimination in pay, work practices, and career advancement*:

- Employment contracts are terminated when maternity leave starts. (I29)
- There are many things tied to being a woman, like being belittled. (I50)
- A previous supervisor doctor who was responsible for training once said to me, You’re reaching the age when you’ll start having children, and then your specialization plans will get sidelined. (I20)

*Lack of integrity and courage*

*Leaders avoiding responsibility*:

- [The leader] covers up their own laziness … fumble around to escape all responsibilities. (I27)

*Insufficient leadership presence, especially where junior physicians require guidance*:

- When a young doctor first starts out … they’re … expected to work just like someone who’s been doing the job for 20 years. (I45)

*Bullying and humiliation*

*Supervisors engaging in psychological harassment, belittling and public criticism*:

- I’ve personally been bullied by my supervisor. (I36)
- They could mercilessly put down those at the bottom of the medical hierarchy if they noticed that someone didn’t quite understand. (I37)
- In front of everyone else, … was criticized and humiliated. (I26)
- Doctor is put against the wall and critiqued in front of the whole clinic. (I35)

*Aggressive behaviour toward subordinates*:

- I corrected him [the leader], and he got upset, slammed his fist on the table, and told me to get the hell out of here. (I15)

CONSEQUENTIALISM (focus on outcomes of actions):

- Negative consequences for well-being (overwork, distress, burnout), turnover and resignations, mistrust and reduced morale, risk to patient care due to inadequate supervision, organizational instability.

*Harmful consequences for staff well-being*

*Overwork, burnout, and emotional distress caused by poor leadership*:

- People aren’t often thanked, once someone leaves, it’s just, Oh, they left, let’s find someone new. (I14)
- There was no supervision offered. (I24)
- The supervising doctor was at another centre many kilometres away, available only by phone. (I24)
- In job interviews, they ask if you plan to have children… It’s clear that you only get a permanent position after having kids. (I50)

*Bullying and humiliation leading to psychological harm*:

- Experiences of injustice… for instance, after a tough night shift, harsh feedback might be given. (I35)
- Doctor is put against the wall and critiqued in front of the whole clinic. (I35) A decision comes out of the blue, stating that such a decision was made two weeks ago. (I36)
- If something was done behind their [leaders] back, they would lash out completely… It is essentially psychological harassment. (I37)

*Negative organizational consequences*

*Physicians leaving their jobs due to poor leadership*:

- A colleague voted with their feet and left, and I realized that if she was leaving, I wouldn’t stay either. (I14)

*Accumulated dissatisfaction leading to group resignations and loss of experienced physicians (I42)*:

- The head of that large company had a negative attitude toward doctors… especially towards experienced doctors. It led to a situation where our concerns weren’t heard… it made the older doctors really angry and eventually led them to leave and I left too. (I42)

*Deterioration of trust and morale*

*Secretive decision-making eroding trust*:

- I’ve seen a lot of that kind of ‘slippery’ leadership, where only the bare minimum leadership role is performed, just administrative decisions are made, and with little effort, while the focus is on other matters. (I35)

*Perceived injustice decreasing motivation and loyalty*:

- In May, I was informed that I couldn’t take any summer vacation. (I49)

*Compromised learning and patient care environment*

*Junior physicians working without adequate supervision, generating potential risks*:

- I just felt like this is what working as a doctor is like, you’re doing it on your own without any leadership. There’s a nominal chief doctor at another health centre, whom you see maybe once a month in a meeting, and they occasionally call to check how things are going … Leadership should be present, it should be accessible. (I24)
- When a young doctor first starts out …, without considering their capabilities … You have to take into account people’s abilities and endurance. (I45)

*Leaders’ refusal to listen or provide guidance*:

- I think that if your subordinate has a problem, you can’t just brush it off with an irresponsible response like, I don’t know what to do, good luck, goodbye. (I34)

DEONTOLOGICAL ETHICS (Focus on duties, moral rules, respect for persons, equality):

- Violation of duty to treat individuals as ends in themselves, inequality and unfair treatment, breach of procedural fairness, failure to uphold supervisory duties, gender discrimination, failure to ensure fair working conditions and contract rights.

*Violation of duty to respect persons*

*Treating people as means rather than ends*:

- But then I heard through the grapevine that they [the leaders] had discussed they could have had four doctors there [instead of the two of us] … Well, go ahead and hire four now. (I14)
- There was this physician working at the health centre who felt they had too much work. They told the boss at the time and wanted to reconsider their job role … The person left after that. (I14)
- But maybe, when thinking about the physician who left our workplace, it feels more like, even though no one is irreplaceable. (I14)

*Dismissal and belittlement based on gender or maternity. Preferential hiring and concealed recruitment processes*:

- I’ve also been asked in an interview, As a married woman, how do you plan to manage this job? (I29)
- I thought, no way, my career can’t be derailed just because I’m a woman. (I20)
- You can also see clearly that if I suggest something regarding a procedure or a patient, it’s only taken seriously when a male doctor suggests it. (I50)

*Humiliation and disrespect*:

- I went to have a discussion with [the leader], but what ended up happening was that in front of everyone else, me, who brought up the problem, was criticized and humiliated. (I26)

*Unjust treatment after night shifts*:

- Harsh feedback might be given when reviewing the cases handled during the night. (I35)

*Failure to uphold duties of fairness and equality*

*Unequal salaries without justification and refusal to acknowledge the issue*:

- I had a pretty unpleasant experience myself. I went to present to the chief physician, as there were two of us [physicians] in the same position, and I knew the other person had a higher salary than I did … I made an appointment, and when I raised the issue, he responded, ‘Well, the other physician has a PhD’ I replied that a PhD is not linked to the base salary, even though it gives a 6% bonus…corrected him, and he got upset. (I15)

*Unequal treatment of women in career progression*:

- Gender disparity still exists … not only in terms of pay but also in terms of work discrimination, like with maternity leave. (I29)
- It felt extremely unfair to use my gender and potential motherhood as a reason. (I20)
- I’ve always thought that if a position is open, it’s advertised in the medical journal or on the employment office’s website … But it still sometimes works like this … they apply, and then the posting is taken down before anyone else can apply. Then, of course, the person who was lined up gets the job. (I29)

*Failure to follow procedural duties*

*Decisions made without proper consultation*:

- All of a sudden, there’s just an email saying that this will now be done this way, without any form of discussion. (I36)

*Lack of thorough information-gathering before criticism*:

- The chief physician had heard about an on-call situation from the nurses. Then they approached me in the cafeteria and scolded me about the situation without ever asking me what had actually happened. In think that in a problematic situation, all parties should be heard. (I34)

*Denial of legally and contractually expected vacation*:

- I was informed that I couldn’t take any summer vacation … It felt unfair … I thought, this can’t be real, this can’t be how it works … I think they even said they’d pay it out in cash, that I’d just keep working until the end of August and then get the vacation money. (I49)

*Neglect of duty of care for subordinates*

*Failure to support physicians after traumatic clinical events. Leadership absence despite supervisory responsibilities*:

- I encountered a situation for the first time where my patient committed suicide … I turned to the acting supervisor … His response was, Well, I don’t know what to tell you, I’ve never had a patient commit suicide. (I34)

*Downplaying employees’ distress*:

- There was no supervision offered. It was more like, Well, better luck next time, things will go smoother next time.” (I24)

*Source:* Authors’ own work

## Discussion

This study aimed to examine, through a deductive content analysis of semi-structured interviews with Finnish physicians (*n* = 50), how poor physician leadership violates the principles of virtue ethics, consequentialism and deontological ethics (Doukas *et al.*, 2022; Everett *et al.*, 2018; Tseng and Wang, 2021). The results indicate that poor leadership contradicts the core tenets of all three ethical frameworks. It was characterized by deficiencies in professional virtues (e.g. lack of transparency, empathy and integrity, including instances of public humiliation and bullying); outcomes that were harmful to staff welfare and organizational functioning (e.g. psychological distress, resignations and reduced trust); and breaches of duty-focused norms and fair procedures (e.g. opaque decision-making, inequitable hiring and pay and inadequate supervisory support). The following sections contextualize these findings within the existing literature, emphasizing leadership behaviours that undermine fairness, respect and support in health-care settings.

### Leadership behaviours, burnout and retention

According to the findings of this study, violations of normative ethics in physician leadership are associated with negative personnel outcomes, such as psychological distress, resignation and staff turnover. This aligns with Antonakis’s assertion that “quality of life depends on the quality of leadership” (Antonakis, 2011). It is also supported by a recent multicentre cross-sectional survey, indicating that physicians who rated their supervisors’ leadership higher reported substantially lower odds of burnout and a lower intention to leave their organizations, suggesting that leadership quality is a strong predictor of physician well-being and retention (Mete *et al.*, 2022).

### Workplace mistreatment, discrimination and occupational harm

Furthermore, bullying, public humiliation, gender-based differential treatment and discrimination are consistent with systematic evidence showing that mistreatment in clinical training and practice settings is common and strongly associated with adverse mental health outcomes. Large-scale surveys of trainees and clinicians have linked experiences of discrimination, abuse, and harassment to increased burnout and suicidal ideation, particularly among women and other disadvantaged groups. These findings support the ethical concern highlighted in our study, as abusive or discriminatory leadership practices are not only morally problematic but also empirically linked to clinician harm (Hu *et al.*, 2019).

### Virtue ethics and professional formation in health care

The absence of reported examples of virtuous leadership (e.g. transparent, empathic and integrity-based supervisory conduct) is consistent with recent scholarship calling for the reinvigoration of virtue ethics in quality improvement and professional formation (Verstegen *et al.*, 2024). Contemporary literature suggests that cultivating professional virtues and practical wisdom (phronesis) complements structural and rule-based quality initiatives, while organizational cultures that neglect virtue cultivation risk undermining the moral dimensions of care (Verstegen *et al.*, 2024). The findings reveal frequent character-related leadership failings, supporting virtue ethics perspectives that organizational context and leadership behaviour are central to clinicians’ moral formation and everyday ethical practice (Verstegen *et al.*, 2024).

### Virtue-ethical perspective

From a virtue-ethical standpoint (Doukas *et al.*, 2022), the findings of humiliation, bullying, evasive leadership and the neglect of subordinate welfare reflect a deficit of core professional virtues (e.g. honesty, compassion, justice and practical wisdom) in the leadership behaviours described by informants. Virtue ethics emphasizes habituation, role modelling and organizational culture as essential for virtue formation. Therefore, persistent patterns of nonethical leadership are problematic not only for discrete interactions but also for the socialization of novices and the moral ecology of work units. Virtues can be acquired through practice and through direct engagement with others (Verstegen *et al.*, 2024). Consequently, demoralization and staff departure, reflecting the absence of virtuous exemplars, may undermine moral development and reduce intrinsic motivation for ethical practice.

### Consequentialist perspective

Consequentialist evaluation focuses on aggregate outcomes (Everett *et al.*, 2018). The interview data reveal recurrent negative consequences of leadership behaviour, including psychological distress, inadequate support following critical incidents, turnover and deterioration in working conditions. Accordingly, suboptimal leadership is linked to higher burnout and a greater intention to leave, which is consistent with previous literature indicating lower burnout and reduced turnover under supervisors who exhibit more favourable leadership behaviours (Mete *et al.*, 2022). From a consequentialist standpoint (Everett *et al.*, 2018), the leadership practices perceived by informants would be considered morally deficient because they are described as producing harmful effects for clinicians and, by extension, may also compromise patient safety and organizational performance. These effects may extend further to broader societal consequences, including reduced quality of patient care and potential erosion of public trust in health-care systems.

### Deontological perspective

Findings of non-transparent decision-making, ad hoc or preferential hiring and inequitable contract practices align with deontological ethics (Tseng and Wang, 2021) emphasizing duties, rights, and procedural fairness. Informants reported a lack of due process in hiring and promotion, inequitable pay treatment and failures to fulfil supervisory obligations, all of which indicate breaches of procedural and interactional duties owed to staff. Research on organisational justice underscores the importance of procedural fairness and respectful interactions for employee well-being and organizational legitimacy (Magnavita *et al.*, 2022). Therefore, the findings of our study align with a deontological critique of leadership practices that instrumentalize physicians or treat them merely as means to administrative ends (Mieth and Williams, 2023).

### Practical and theoretical implications

The findings reveal deficiencies in ethical physician leadership and underscore the practical need to strengthen education in virtue ethics, consequentialist sensitivity to outcomes and deontological commitments to fairness within medical leadership curricula and organizational policy. Interventions targeting leadership behaviours could plausibly mitigate both moral failings and their associated adverse outcomes. The study highlights the potential of leadership development and well-being-centred leadership to reduce clinician burnout and turnover intentions. Virtue-based educational approaches, combined with an organizational emphasis on procedural justice, can serve as effective strategies for enhancing professional culture and care quality, as recent scholarship suggests that virtue formation (e.g. fostering practical wisdom and empathic supervision) may enrich quality improvement and professional formation programmes (Verstegen *et al.*, 2024). Concurrently, organizations should remain attentive to outcome metrics (e.g. burnout and retention) as well as procedural safeguards that uphold deontological commitments to fairness and respect. Promoting transparent decision-making, strengthening supervisory support structures following critical incidents and implementing clear and equitable human resources procedures are interventions supported by both our data and the literature on organizational justice and leadership. Leadership development programmes could be further strengthened through case-based learning grounded in real ethical dilemmas, feedback focused on ethical leadership behaviours and structured training in procedural justice for physician leaders. Taken together, these approaches reflect the need for a pluralist normative framework that recognizes the complementary moral insights of virtue ethics, consequentialism and deontology in both research and practice on physician leadership.

## Conclusion

The results portray leadership practices that violate the evaluative standards of virtue ethics, consequentialism and deontological ethics. These findings highlight ethical vulnerabilities in leadership practices within the examined health-care setting, underscoring the need to reinforce ethical principles in both leadership training and organizational governance.

## Data Availability

The data that support the findings of this study are available from the corresponding author upon reasonable request.

## Appendix. Research questions

How would you describe a good physician leadership?

Does a leader of physicians need to be a physician her/himself?

Additional research questions for leaders

What kind of leader are you?

What career path have you taken to your current position, and what does your job entail?

How would you define/explain physician leadership to someone who is unfamiliar with the concept?

What key aspects would you highlight as the most important in physician leadership?

Is physician leadership different from other types of leadership?

What areas do you focus on in your own leadership work?

- filling out the leadership role form.

What aspects of your leadership do you find:

- Easy/rewarding/positive?
- difficult/challenging/stressful?

How could physician leadership work be supported?

What are your thoughts on the current leadership training for physicians?

How do you feel about balancing your work and personal life?

Is there anything else you would like to share about physician leadership?
